# HiChIPdb: a comprehensive database of HiChIP regulatory interactions

**DOI:** 10.1093/nar/gkac859

**Published:** 2022-10-10

**Authors:** Wanwen Zeng, Qiao Liu, Qijin Yin, Rui Jiang, Wing Hung Wong

**Affiliations:** Department of Statistics, Stanford University, Stanford, CA 94305, USA; Bio-X Program, Stanford University, Stanford, CA 94305, USA; College of Software, Nankai University, Tianjin 300350, China; Department of Statistics, Stanford University, Stanford, CA 94305, USA; Bio-X Program, Stanford University, Stanford, CA 94305, USA; Ministry of Education Key Laboratory of Bioinformatics, Bioinformatics Division at the Beijing National Research Center for Information Science and Technology, Center for Synthetic and Systems Biology, Department of Automation, Tsinghua University, Beijing 100084, China; Ministry of Education Key Laboratory of Bioinformatics, Bioinformatics Division at the Beijing National Research Center for Information Science and Technology, Center for Synthetic and Systems Biology, Department of Automation, Tsinghua University, Beijing 100084, China; Department of Statistics, Stanford University, Stanford, CA 94305, USA; Department of Biomedical Data Science, Stanford University, Stanford, CA 94305, USA; Bio-X Program, Stanford University, Stanford, CA 94305, USA

## Abstract

Elucidating the role of 3D architecture of DNA in gene regulation is crucial for understanding cell differentiation, tissue homeostasis and disease development. Among various chromatin conformation capture methods, HiChIP has received increasing attention for its significant improvement over other methods in profiling of regulatory (e.g. H3K27ac) and structural (e.g. cohesin) interactions. To facilitate the studies of 3D regulatory interactions, we developed a HiChIP interactions database, HiChIPdb (http://health.tsinghua.edu.cn/hichipdb/). The current version of HiChIPdb contains ∼262M annotated HiChIP interactions from 200 high-throughput HiChIP samples across 108 cell types. The functionalities of HiChIPdb include: (i) standardized categorization of HiChIP interactions in a hierarchical structure based on organ, tissue and cell line and (ii) comprehensive annotations of HiChIP interactions with regulatory genes and GWAS Catalog SNPs. To the best of our knowledge, HiChIPdb is the first comprehensive database that utilizes a unified pipeline to map the functional interactions across diverse cell types and tissues in different resolutions. We believe this database has the potential to advance cutting-edge research in regulatory mechanisms in development and disease by removing the barrier in data aggregation, preprocessing, and analysis.

## INTRODUCTION

3D genome architecture is central to the complex gene regulation but challenging to interrogate (1–4). Rapid advances in next-generation sequencing technologies have provided an unprecedented opportunity to investigate 3D genome interactions on a genome-wide scale. Among various chromatin conformation capture (3C)-based methods, HiChIP (5) has received increasing attention for its significant improvement over ChIA-PET (6) and Hi-C (7) in direct profiling of regulatory (e.g. H3K27ac) and structural (e.g. cohesin) interactions (8). The novel protein-mediated high-throughput sequencing technique generates high-quality and high-resolution contact maps of genome spatial organization while lowering cellular input requirements. Recent years have witnessed a surge of HiChIP assays across diverse cell lines that characterize functional relevance of chromatin interactions and provide extraordinary insights into diseases arising from mutations affecting epigenetic modifiers, transcriptional factor binding loci, and architectural proteins (9). However, most publicly available HiChIP data remain underutilized as little effort has been made to assemble a comprehensive collection of HiChIP samples scattered in literature. Existing 3D databases, such as ENCODE (10) and 4D Nucleome (11) and others (12–14), feature mostly Hi-C data and fail to incorporate HiChIP assays. The built-in Hi-C-specific preprocessing tools found in these databases are also not optimal for HiChIP data preprocessing. More importantly, most 3D databases focus heavily on interaction visualization which is essential for Hi-C but tend to overlook the functional implications of protein-directed HiChIP samples. Moreover, the public HiChIP data are being accumulated at an unprecedented rate: of the 247 human HiChIP datasets in GEO repository, 85 (34.4%) were generated in the past 12 months. The rapid growth rate of HiChIP data indicates the potential impact on a broad research community to leverage these types of 3D functional interactions to study regulatory mechanism. Therefore, to facilitate the studies of 3D regulatory interactions and promote comprehensive analysis of HiChIP data, we developed HiChIPdb (http://health.tsinghua.edu.cn/hichipdb/), a comprehensive database of HiChIP data.

**Figure 1.**
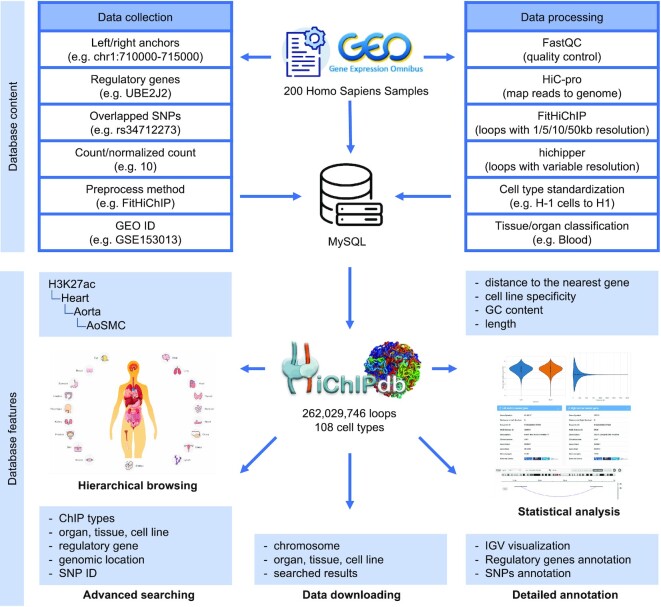
Overview of data collection, data processing and annotation, and database features of HiChIPdb.

In total, HiChIPdb currently contains ∼262M annotated HiChIP interactions from 200 high-throughput HiChIP samples across 108 cell types with multi resolutions. Under 5 kb resolution, 2 142 294 loops spanning 496 763 anchors were observed among all samples respectively (Figure 1). All HiChIP samples are preprocessed using a unified framework including FastQC basic read quality check, HiC-Pro alignment, quality control and replicate merge (15). Due to the low read-pair density in HiChIP data, multiple resolutions at 1/5/10/50 kb are offered through the computational tool FitHiChIP (16), and variable length resolution through hichipper (17). Each HiChIP sample in HiChIPdb is annotated hierarchically with extensive details such as cell line, tissue, organ, etc. Various statistical measures including the length distribution of HiChIP anchors, the length distribution of HiChIP loops, and the density distribution of HiChIP interactions in different chromosomes are provided on the Browse page for cross-referencing. Unlike Hi-C and other conventional chromatin conformation strategies, HiChIP signals indicate protein-centric *in situ* chromatin loops that carry significant functional implications. In addition to the above-mentioned visualization tools, HiChIPdb has a strong emphasis on functional annotations of regulatory genes and GWAS catalog SNPs overlapping with HiChIP anchors. Annotated genes are listed on the *Detail* page of respective interactions with links to external databases (e.g. GeneCards (18), UniProt (19) and NCBI (20,21), etc.). Similarly, annotated SNPs are linked to dbSNP (22). Other functionalities in HiChIPdb include advanced searching, hierarchical browsing, interactive visualization with custom tracks and data downloading with different options. A detailed preprocessing and statistical analysis of a HiChIP sample is presented as an exemplary usage of the database on the *Tutorial* page.

Overall, HiChIPdb presents a comprehensive compilation of published HiChIP samples with reliable annotations and user-friendly interfaces. In addition to its collection of informative data, HiChIPdb is developed to ultimately benefit prospective downstream applications. Before the development of this database, several studies have already started to leverage protein-centric HiChIP data to computationally model genomic architecture (23–25). Despite this, systematic efforts to fully leverage HiChIP data across numerous samples remain elusive. HiChIPdb aims to address this challenge and offers a promising avenue for researchers to investigate genome-wide chromatin interactions across different cell types in the context of various proteins of interest. Many advantages of HiChIPdb, such as a stringent preprocessing pipeline with multiple resolution options, extensive annotations of epigenomic profile and GWAS catalog (26), are designed to facilitate integrative analysis and/or modeling of various properties of 3D chromatin structure. We anticipate this database to empower future computational approaches and deepen our understanding of gene regulatory networks and disease mechanisms.

## DATA COLLECTION AND DATABASE CONTENT

### Data collection

To collect HiChIP datasets, we referred to a series of standardized procedures of biological databases for consistent and reliable data collection. First, we searched the keyword ‘HiChIP’ in Gene Expression Omnibus (GEO) database (20,21) (https://www.ncbi.nlm.nih.gov/geo/) and retrieved 989 results (as of May 2022) with each result corresponding to a candidate HiChIP dataset. Second, these candidate HiChIP datasets were filtered on the availability of raw SRA sequencing data, reducing the total number of datasets to 247. Validated HiChIP datasets were identified by providing at least one sample of a specific human tissue or cell line. Each dataset contains general information such as cell type, treatment, ChIP type, GEO accession. We manually extracted the information of tissue and organ based on cell type to construct a hierarchical structural tree of the curated database (Figure 1).

### Data processing and annotation

In order to make the HiChIP data comparable across different tissues and cell types, we proposed here a unified framework for data processing for each HiChIP dataset. First, we processed each HiChIP dataset from the SRA raw sequencing data (e.g. FASTQ) and produced HiChIP interactions at various resolutions with detailed annotations through the unified data processing pipeline (Figure 1). Specifically, we first applied HiC-Pro (15) software for processing raw FASTQ files (paired-end Illumina data), including reads mapping, valid ligation products detection, quality control and intra/inter-chromosomal contacts map generation. We then chose GRCh37/hg19 as the reference genome for Homo sapiens, and applied FitHiChIP (27) and Hichipper (17) for HiChIP data loop calling. Note that FitHiChIP enables loop calling at specific resolutions (1k, 5k, 10k and 50k), which resulted in fixed loop size across the whole genome. In contrast, Hichipper produces variable-length loops, which makes it difficult to compare across cell types. HiChIP samples with less than 100 loops were filtered for quality control. The current release (as of May 2022) of HiChIP database contained 200 HiChIP samples from 64 GEO repositories. After standardizing the names of cell types into the standard list from ENCODE (28) and removing the ill-formed names, we further categorized each HiChIP dataset into respective cell type, tissue and organ to form a hierarchical structure.

Finally, to further facilitate the study of 3D genome regulatory mechanisms, each loop was annotated with a rich set of features, including the nearest genes to each loop anchor, Genome-Wide Association Studies (GWAS) Catalog SNPs (26) within the loop anchors, the raw/normalized read count within the loop anchors and the p-value for the loop. Each loop was visualized using IGV interactive tool (29) and the reference genes within the loop range were annotated on the illustration.

### Database statistics

The current release of HiChIPdb contains 200 high-throughput HiChIP samples across 108 cell types, 19 organs and 28 tissues. Under 5 kb resolution, 21 422 946 loops spanning 496,763 anchors were observed among all HiChIP samples. Among the 12 different ChIP-associated proteins, H3K27ac is the dominant protein that covers 17,612,271 (82.21%) of the full HiChIP chromatin loops as H3K27ac HiChIP directly identifies high-confident functional loops focused around enhancer interactions (5). There are also 355,968 HiChIP loops (1.66%) associated with cohesion protein, which reveal multi-scale genome architecture (5). The total number of HiChIP loops was approximately 6.5M, 29.5M and 28.4M when we set the resolution to 1k, 10k and 50k, respectively. We also notice that the number of HiChIP loops from Hichipper is about 176M, which is much larger than FitHiChIP-based pipeline. The H3K27ac-associated HiChIP loops are mainly from blood (31.58%), brain (17.01%) and skin (11.51%) tissues.

## DATABASE FEATURES

### User-friendly browsing

**Figure 2.**
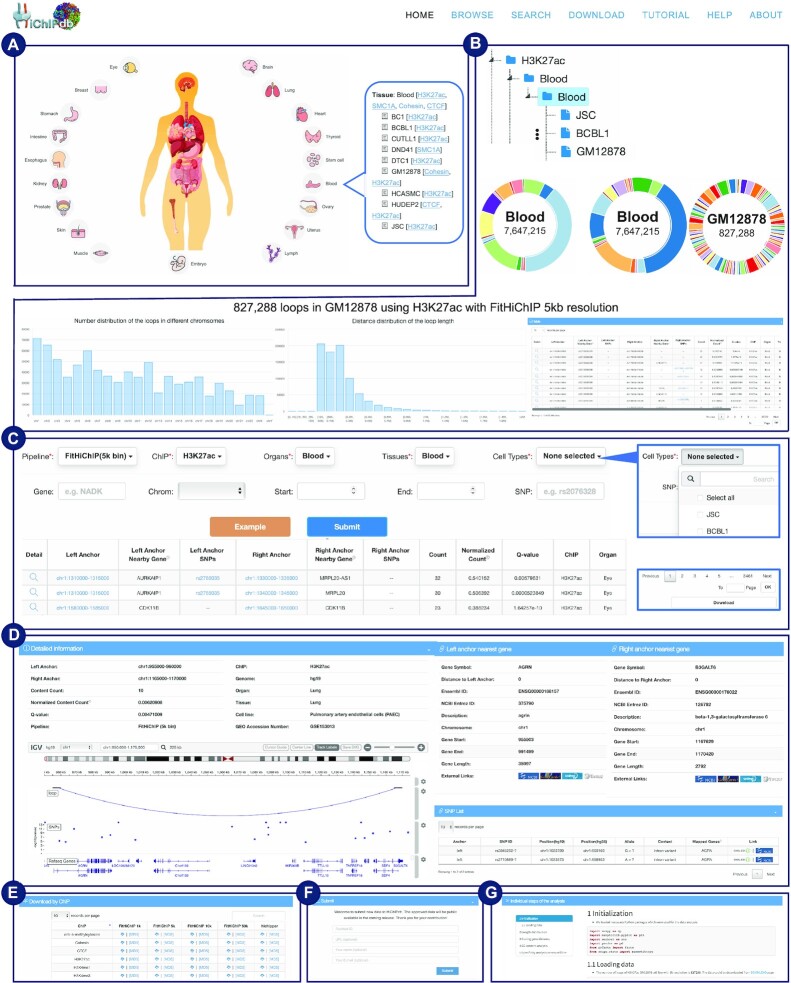
The schematic features on various webpages of HiChIPdb. (**A**) *Home:* interactive images of human anatomy (**B**) *Browse*: hierarchical categorization of HiChIP interactions and comprehensive statistics for the selected subset of interactions. (**C**) *Search*: various options and filters for searching the interactions of interest. (**D**) *Detail:* detailed information of each interaction entry (**E**) *Download*: multiple standard-compliant download options with external links. (**F**) *About*: HiChIP dataset submission portal. (**G**) *Analysis*: an exemplary case study of HiChIP interactions in GM12878.

We built an interactive web interface for researchers to explore the well-organized HiChIP data across diverse cellular contexts. An interactive image of human anatomy displayed on the *Home* page enables direct access to HiChIP loops related to a specific human organ (Figure 2A). In brief, we make it easy for users to access relevant cell lines within a pop-up window by clicking one of the organ icons. Alternatively, users can also directly explore the HiChIP datasets in a tree-based hierarchical order (ChIP-Organ-Tissue-Cell type) through the *Browser* page (Figure 2B). Users can select a specific ChIP (e.g. H3K27ac) in the left panel tree structure through a unified pipeline (default is 5k resolution). Once selected, a set of comprehensive statistics will be illustrated through pie plots or bar plots which include: (i) distribution of the loops number across various organs; (ii) distribution of the loops number across various tissues; (iii) distribution of the loops number across various cell types; (iv) distribution of the loops number across different chromosomes; (v) distance distribution of the loop length (within 2M bp). In addition, an interactive table is generated, where each row represents a HiChIP loop and columns denote the detailed information including anchor location, anchor nearest gene, SNPs within the anchors, raw/normalized read count within the anchors, cell type, tissue, organ, and the GEO accession number (Figure 2C). Clicking on the ‘Detail’ button generates a visualization for each loop, and includes the reference genes within the loop region (Figure 2D).

### Advanced searching

On the *Search* page, various options in the drop-down menus allow for searching HiChIP loops that meet specific conditions (Figure 2C). Users can choose different conditions including pipeline type, ChIP type, organs, tissue and cell types. Note that multi-select and select-all are enabled for organs, tissues, and cell types. An example button is provided for users to quickly select a combination of filtering conditions. Users can simply click the submit button to get the filtered HiChIP loops in the result table. A download option is also available for users to download the filtered HiChIP loop table of results.

### Detailed information

Detailed information on each HiChIP loop is available through the *Browse* or *Search* page, and each HiChIP loop is linked to the *Detail* page of the entry (Figure 2D). The redirected *Detail* page will then demonstrate various information about the chosen HiChIP loop, including interactive visualization, anchor information, nearby gene, and SNPs within the loop region. The interactive visualization reports the loop, SNPs, *P*-values of the SNPs and RefSeq genes, with zoom in/out option. Users can also specify multiple samples of interest for further comparison and investigation. The *Detail* page also provides overviews of the annotated nearby genes with gene symbols, chromosomes, start and end sites. More information about the genes can be available through external links, including NCBI (20,21), GeneCards (30), UniProt (19). As the genetic variants may affect the chromatin contact propensity, thus potentially changing the gene expression (31–33), we also provided external EMBL-EBI (34) and NCBI (20,21) links for all the SNPs that fall into the HiChIP loop anchors.

### Data download and submission

Various options are available for users to download HiChIP data by ChIP, chromosome, organ, tissue, cell type and sample through the *Download* page (Figure 2E). An MD5 checksum is included for each downloaded file for verifying the integrity. For each download table with a specific option (e.g. ChIP), users can search for a specific name to quickly locate the file with interest using the searching box. To make HiChIPdb scalable, a submission window on the *About* page allows users to add their newly generated HiChIP data to the HiChIPdb by submitting the GEO accession number, URL for data accession, and contributing authors information (Figure 2F). After approval, the dataset is then automatically deployed to the HiChIPdb web server through a standardized pipeline, and the processed data will be integrated with existing HiChIP datasets.

### The uniqueness of HiChIPdb compared to other 3D genome databases

There are currently only four existing 3D genome databases including 4DN (11), 3D genome browser(3DGB) (12), HUGIN2 (not published) and 3DIV (14). To the best of our knowledge, HiChIPdb is the first comprehensive 3D genome database that utilizes a unified pipeline to provide functional interaction data across diverse cell types and tissues. The major differences between HiChIPdb and other 3D genome databases are demonstrated in the following three aspects (Table 1). First, HiChIPdb provides the most comprehensive HiChIP functional loops resource while most databases ignore HiChIP data except HUGIN2. However, HUGIN2 only contains 12 HiChIP datasets without any functional annotation. Other comparing databases mainly focus on Hi-C data and do not provide detailed information of uniformed loops, loop anchors, and loop functional annotations, which are highlighted features of HiChIPdb. As a resource for computational biologists, HiChIPdb provides various export options on the *Search* and *Download* page for downstream analysis. Second, HiChIPdb is the only resource that uses a unified pipeline with multiple optional resolutions, thus making it possible to compare the HiChIP functional loops across cell types. To systematically analyze 3D functional genome, the unified strategy is important for calculating the cell type specificity of functional loops and investigating the interactions of functional regulatory elements in whole genome. Third, Our HiChIP database provides various functional annotations (e.g. regulatory genes, GWAS Catalog SNPs, etc.), which can facilitate diverse research related to functional genome studies.

**Table 1.** Comparison of HiChIPdb to other 3D genome databases, including 4DN, 3D genome browser (3DGB), HUGIN2 and 3DIV. Most 3D genome databases mainly focus on Hi-C data while only HiChIPdb and HUGIN2 incorporate HiChIP data

Function type	Data type/specific function	HiChIPdb	4DN	3DGB	HUGIN2	3DIV
Data coverage	Contain HiChIP data	√			√	
Data browser	Simple information browser	√	√	√	√	√
	Hierarchical data organization	√				
	Table browser	√	√		√	√
Visualization	Interactive data entry	√	√			
	Interactive statistics	√				
	Loop visualization	√				√
Data search	By genomic region	√		√	√	√
	By gene	√		√	√	√
	By SNP	√		√	√	√
Annotation	Nearby gene	√		√	√	√
	GWAS Catalog SNPs	√			√	√
	Loop anchor information	√				
Download	By sample	√	√			√
	By tissue	√				
	By organ	√				
	By chromosome	√				
	File hash check	√				

### A tutorial of HiChIPdb

To make HiChIPdb easy to use, a comprehensive tutorial on how to analyze processed HiChIP data and how to apply the processed HiChIP data in downstream applications is provided on the *Tutorial* page. Useful statistical information of the loops is shown for an example dataset. For instance, for the 827,288 HiChIP loops with 5k resolution from GM12878 cell line, the length of loops ranges from 20 kb to 2 Mb, and the median distance between the left loop anchor and right loop anchor to the nearest gene is 39 457 bp and 39 830 bp, respectively. The GC content of the left loop anchor and right loop anchor is both 0.02–0.74, which demonstrates little difference between anchors. The tutorial also explores cell type specificity of the loops from HiChIP CTCF experiments of GM12878 cell line. Some loops only exist in a few cell lines while other loops exist in almost all of the cell lines, demonstrating cell type specificity of HiChIP loops.

The tutorial also demonstrates the effectiveness of HiChIPdb through the application of annotating GWAS risk genes by using HiChIP loops. Given the GWAS summary statistics data, all HiChIP anchors that contain at least one GWAS SNP are collected. Here, $( { - {{\log }_{10}}P} ) \times \beta$ is defined as the weighted effect size for each variant where $\beta$ and *P* denote the effect size and *P*-value in the original GWAS study, respectively. And then the weighted effect size of all genetic variants that fall on the regions that have interactions with the target risk gene are summed together as the HiChIP annotated gene score (HAGS). The HAGS describe the accumulated effect of all GWAS SNPs with implicated interaction with the risk gene. Different from merely sum up the effect of SNPs that are nearby the target gene. HAGS provide a more comprehensive measurement of the overall genetic variants effect on a specific target gene.

A QT interval duration study (35) is taken as an illustrative example. HAGS for all genes across different cell types using H3K27ac HiChIP interactions are calculated. Interestingly, two risk genes (NOS1AP and KCNH2) reported in (35–37) were ranked 1st and 12th in HAEC heart cell line, respectively. In contrast, NOS1AP ranked 3rd while KCNH2 ranked 11 729th in PAEC lung cell line, NOS1AP and KCNH2 all ranked 4179th in HARA lung cell line due to no overlapped HiChIP interactions. The mean ranking and median ranking for these two genes in irrelevant cell lines are further calculated as background ranking. NOS1AP has a mean rank of 2229th, median rank of 470th in heart irrelevant cell lines. And KCNH2 has a mean rank of 2093th, median rank of 280th in heart irrelevant cell lines. Such results demonstrate that the HAGS is a useful statistic for discovering potential disease-associated genes.

## SYSTEM DESIGN AND IMPLEMENTATION

The HiChIPdb website is developed and maintained on a Linux Apache web server (https://www.apache.org). The frontend interface of our database uses Bootstrap v3.3.7 framework (https://getbootstrap.com/docs/3.3/) for display. Plug-ins for the JavaScript and jQuery libraries, including Biodalliance v0.13.8 (http://www.biodalliance.org/about.html), DataTables v1.10.19 (https://datatables.net), and morris.js v0.5.0 (https://morrisjs.github.io/morris.js/index.html), are used for implementing advanced tables and charts, respectively. The backend of the server uses PHP v7.4.5 (http://www.php.net). All the processed data are stored in a MySQL v8.0.20 (http://www.mysql.com) database. The current version of HiChIPdb supports most of the standard web browsers, including Google Chrome, Apple Safari, etc.

## CONCLUSION

The investigation of transcriptional regulation is one of the most influential areas of life science research. Using the HiChIPdb allows investigators to interpret the underlying mechanisms of regulatory interactions systematically due to the advancements in both experimental and computational techniques. Prior analytical methods have been largely restricted to studying 3D genome using 3C-based technology such as Hi-C (38–40), with less emphasis given to functional interactions such as enhancer-promoter interactions (41). Recent breakthroughs in 3D functional interactions research, such as HiChIP, brings these functional interactions into the spotlight (24,25,42), allowing investigators obtain additional insight into the spatial and temporal dynamics of gene regulation that was not possible before. However, systematic efforts to provide uniformly processed and fully annotated HiChIP functional 3D interactions across diverse samples remain lacking using prior databases.

To fill the gap, we developed HiChIPdb, a comprehensive database that focuses on HiChIP regulatory interactions. HiChIPdb uses systematic data pre-processing procedures and has a user-friendly web platform including advanced searching, interactive visualization and convenient download. HiChIPdb can assist biologists and data scientists achieve a better understanding of the role of functional interactions in gene regulatory mechanisms and empower them to construct more comprehensive gene regulatory networks. For example, since GWAS-identified risk variants in non-coding regions of the genome exert phenotypic effects through perturbation of functional 3D interactions, HiChIPdb has the potential to give insights to a more complete interpretation of GWAS risk variants and aid in developing new approaches for disease prevention and treatment (43–45).

To make HiChIPdb more comprehensive and useful, we plan to incorporate the following features in our future release. The first thing is to collect mouse samples and integrate them into HiChIPdb, which can be supplement for human samples to better understand regulatory mechanism. Second, we plan to incorporate more comprehensive epigenomic annotations, such as different types of transcription factor binding, histone modification and chromatin accessibility annotation for each HiChIP sample. Third, to improve the usefulness of our database, we would like to build a webserver for fast annotation of functional HiChIP loops with user-specific multiple genomic regions as input. Last but not least, in order to facilitate the collection process and expand our database, we would also like to incorporate a web-based tracking and data entry system to carry out a monthly GEO search for a regular HiChIPdb update.

## DATA AVAILABILITY

HiChIPdb is publicly accessible for worldwide users without any registration or login. Users can freely access all data host in HiChIPdb at http://health.tsinghua.edu.cn/hichipdb.

## References

[1] Gasperini M. , Tome J.M., Shendure J. Towards a comprehensive catalogue of validated and target-linked human enhancers. Nat. Rev. Genet. 2020; 21:292–310. 31988385 10.1038/s41576-019-0209-0 PMC7845138

[2] Andersson R. , Sandelin A. Determinants of enhancer and promoter activities of regulatory elements. Nat. Rev. Genet. 2020; 21:71–87. 31605096 10.1038/s41576-019-0173-8

[3] Chatterjee S. , Ahituv N. Gene regulatory elements, major drivers of human disease. Annu. Rev. Genomics Hum. Genet. 2017; 18:45–63. 28399667 10.1146/annurev-genom-091416-035537

[4] Zheng H. , Xie W. The role of 3D genome organization in development and cell differentiation. Nat. Rev. Mol. Cell Biol. 2019; 20:535–550. 31197269 10.1038/s41580-019-0132-4

[5] Mumbach M.R. , Rubin A.J., Flynn R.A., Dai C., Khavari P.A., Greenleaf W.J., Chang H.Y. HiChIP: efficient and sensitive analysis of protein-directed genome architecture. Nat. Methods. 2016; 13:919–922. 27643841 10.1038/nmeth.3999 PMC5501173

[6] Fullwood M.J. , Liu M.H., Pan Y.F., Liu J., Xu H., Mohamed Y.B., Orlov Y.L., Velkov S., Ho A., Mei P.H. et al. An oestrogen-receptor-alpha-bound human chromatin interactome. Nature. 2009; 462:58–64. 19890323 10.1038/nature08497 PMC2774924

[7] Lieberman-Aiden E. , van Berkum N.L., Williams L., Imakaev M., Ragoczy T., Telling A., Amit I., Lajoie B.R., Sabo P.J., Dorschner M.O. et al. Comprehensive mapping of long-range interactions reveals folding principles of the human genome. Science. 2009; 326:289–293. 19815776 10.1126/science.1181369 PMC2858594

[8] Mumbach M.R. , Satpathy A.T., Boyle E.A., Dai C., Gowen B.G., Cho S.W., Nguyen M.L., Rubin A.J., Granja J.M., Kazane K.R. et al. Enhancer connectome in primary human cells identifies target genes of disease-associated DNA elements. Nat. Genet. 2017; 49:1602–1612. 28945252 10.1038/ng.3963 PMC5805393

[9] Shi B. , Li W., Song Y., Wang Z., Ju R., Ulman A., Hu J., Palomba F., Zhao Y., Le J.P. et al. UTX condensation underlies its tumour-suppressive activity. Nature. 2021; 597:726–731. 34526716 10.1038/s41586-021-03903-7 PMC9008583

[10] Sloan C.A. , Chan E.T., Davidson J.M., Malladi V.S., Strattan J.S., Hitz B.C., Gabdank I., Narayanan A.K., Ho M., Lee B.T. et al. ENCODE data at the ENCODE portal. Nucleic Acids Res. 2016; 44:D726–D732. 26527727 10.1093/nar/gkv1160 PMC4702836

[11] Dekker J. , Belmont A.S., Guttman M., Leshyk V.O., Lis J.T., Lomvardas S., Mirny L.A., O'Shea C.C., Park P.J., Ren B et al. The 4D nucleome project. Nature. 2017; 549:219–226. 28905911 10.1038/nature23884 PMC5617335

[12] Wang Y. , Song F., Zhang B., Zhang L., Xu J., Kuang D., Li D., Choudhary M.N.K., Li Y., Hu M. et al. The 3D genome browser: a web-based browser for visualizing 3D genome organization and long-range chromatin interactions. Genome Biol. 2018; 19:151. 30286773 10.1186/s13059-018-1519-9 PMC6172833

[13] Oluwadare O. , Highsmith M., Turner D., Lieberman Aiden E., Cheng J. GSDB: a database of 3D chromosome and genome structures reconstructed from Hi-C data. BMC Mol. Cell Biol. 2020; 21:60. 32758136 10.1186/s12860-020-00304-y PMC7405446

[14] Yang D. , Jang I., Choi J., Kim M.S., Lee A.J., Kim H., Eom J., Kim D., Jung I., Lee B. 3DIV: A 3D-genome interaction viewer and database. Nucleic Acids Res. 2018; 46:D52–D57. 29106613 10.1093/nar/gkx1017 PMC5753379

[15] Servant N. , Varoquaux N., Lajoie B.R., Viara E., Chen C.J., Vert J.P., Heard E., Dekker J., Barillot E. HiC-Pro: an optimized and flexible pipeline for Hi-C data processing. Genome Biol. 2015; 16:259. 26619908 10.1186/s13059-015-0831-x PMC4665391

[16] Bhattacharya A. , Brea R.J., Niederholtmeyer H., Devaraj N.K. A minimal biochemical route towards de novo formation of synthetic phospholipid membranes. Nat. Commun. 2019; 10:300. 30655537 10.1038/s41467-018-08174-x PMC6336818

[17] Lareau C.A. , Aryee M.J. hichipper: a preprocessing pipeline for calling DNA loops from HiChIP data. Nat. Methods. 2018; 15:155–156. 29489746 10.1038/nmeth.4583 PMC10572103

[18] Stelzer G. , Rosen N., Plaschkes I., Zimmerman S., Twik M., Fishilevich S., Stein T.I., Nudel R., Lieder I., Mazor Y. et al. The genecards suite: from gene data mining to disease genome sequence analyses. Curr. Protoc. Bioinformatics. 2016; 54:1.30.1–1.30.33. 10.1002/cpbi.5 27322403

[19] UniProt C. UniProt: the universal protein knowledgebase in 2021. Nucleic Acids Res. 2021; 49:D480–D489. 33237286 10.1093/nar/gkaa1100 PMC7778908

[20] Barrett T. , Wilhite S.E., Ledoux P., Evangelista C., Kim I.F., Tomashevsky M., Marshall K.A., Phillippy K.H., Sherman P.M., Holko M. et al. NCBI GEO: archive for functional genomics data sets–update. Nucleic Acids Res. 2013; 41:D991–D995. 23193258 10.1093/nar/gks1193 PMC3531084

[21] Edgar R. , Domrachev M., Lash A.E. Gene expression omnibus: NCBI gene expression and hybridization array data repository. Nucleic Acids Res. 2002; 30:207–210. 11752295 10.1093/nar/30.1.207 PMC99122

[22] Sherry S.T. , Ward M.H., Kholodov M., Baker J., Phan L., Smigielski E.M., Sirotkin K. dbSNP: the NCBI database of genetic variation. Nucleic Acids Res. 2001; 29:308–311. 11125122 10.1093/nar/29.1.308 PMC29783

[23] Zeng W. , Wu M., Jiang R. Prediction of enhancer-promoter interactions via natural language processing. BMC Genomics. 2018; 19:84. 29764360 10.1186/s12864-018-4459-6 PMC5954283

[24] Zeng W. , Wang Y., Jiang R. Integrating distal and proximal information to predict gene expression via a densely connected convolutional neural network. Bioinformatics. 2020; 36:496–503. 31318408 10.1093/bioinformatics/btz562

[25] Zeng W. , Chen X., Duren Z., Wang Y., Jiang R., Wong W.H. DC3 is a method for deconvolution and coupled clustering from bulk and single-cell genomics data. Nat. Commun. 2019; 10:4613. 31601804 10.1038/s41467-019-12547-1 PMC6787340

[26] Buniello A. , MacArthur J.A.L., Cerezo M., Harris L.W., Hayhurst J., Malangone C., McMahon A., Morales J., Mountjoy E., Sollis E. et al. The NHGRI-EBI GWAS catalog of published genome-wide association studies, targeted arrays and summary statistics 2019. Nucleic Acids Res. 2019; 47:D1005–D1012. 30445434 10.1093/nar/gky1120 PMC6323933

[27] Bhattacharyya S. , Chandra V., Vijayanand P., Ay F. Identification of significant chromatin contacts from HiChIP data by FitHiChIP. Nat. Commun. 2019; 10:4221. 31530818 10.1038/s41467-019-11950-y PMC6748947

[28] Consortium E.P. A user's guide to the encyclopedia of DNA elements (ENCODE). PLoS Biol. 2011; 9:e1001046. 21526222 10.1371/journal.pbio.1001046 PMC3079585

[29] Robinson J.T. , Thorvaldsdottir H., Winckler W., Guttman M., Lander E.S., Getz G., Mesirov J.P. Integrative genomics viewer. Nat. Biotechnol. 2011; 29:24–26. 21221095 10.1038/nbt.1754 PMC3346182

[30] Rappaport N. , Twik M., Plaschkes I., Nudel R., Iny Stein T., Levitt J., Gershoni M., Morrey C.P., Safran M., Lancet D MalaCards: an amalgamated human disease compendium with diverse clinical and genetic annotation and structured search. Nucleic Acids Res. 2017; 45:D877–D887. 27899610 10.1093/nar/gkw1012 PMC5210521

[31] Albert F.W. , Kruglyak L. The role of regulatory variation in complex traits and disease. Nat. Rev. Genet. 2015; 16:197–212. 25707927 10.1038/nrg3891

[32] Vinuela A. , Varshney A., van de Bunt M., Prasad R.B., Asplund O., Bennett A., Boehnke M., Brown A.A., Erdos M.R., Fadista J. et al. Genetic variant effects on gene expression in human pancreatic islets and their implications for T2D. Nat. Commun. 2020; 11:4912. 32999275 10.1038/s41467-020-18581-8 PMC7528108

[33] Consortium G.T. , Laboratory D.A., Coordinating Center -Analysis Working G., Statistical Methods groups-Analysis Working G., Enhancing G.g., Fund N.I.H.C., Nih/Nci, Nih/Nhgri, Nih/Nimh, Nih/Nida et al. Genetic effects on gene expression across human tissues. Nature. 2017; 550:204–213. 29022597 10.1038/nature24277 PMC5776756

[34] Madeira F. , Park Y.M., Lee J., Buso N., Gur T., Madhusoodanan N., Basutkar P., Tivey A.R.N., Potter S.C., Finn R.D. et al. The EMBL-EBI search and sequence analysis tools APIs in 2019. Nucleic Acids Res. 2019; 47:W636–W641. 30976793 10.1093/nar/gkz268 PMC6602479

[35] Nauffal V. , Morrill V.N., Jurgens S.J., Choi S.H., Hall A.W., Weng L.C., Halford J.L., Austin-Tse C., Haggerty C.M., Harris S.L. et al. Monogenic and polygenic contributions to QTc prolongation in the population. Circulation. 2022; 145:1524–1533. 35389749 10.1161/CIRCULATIONAHA.121.057261 PMC9117504

[36] Aarnoudse A.J. , Newton-Cheh C., de Bakker P.I., Straus S.M., Kors J.A., Hofman A., Uitterlinden A.G., Witteman J.C., Stricker B.H. Common NOS1AP variants are associated with a prolonged QTc interval in the rotterdam study. Circulation. 2007; 116:10–16. 17576865 10.1161/CIRCULATIONAHA.106.676783

[37] Newton-Cheh C. , Guo C.-Y., Larson M.G., Musone S.L., Surti A., Camargo A.L., Drake J.A., Benjamin E.J., Levy D., D’Agostino R.B. Sr Common genetic variation in KCNH2 is associated with QT interval duration: the framingham heart study. Circulation. 2007; 116:1128–1136. 17709632 10.1161/CIRCULATIONAHA.107.710780

[38] Cripps R.M. , Lovato T.L., Olson E.N. Positive autoregulation of the myocyte enhancer factor-2 myogenic control gene during somatic muscle development in drosophila. Dev. Biol. 2004; 267:536–547. 15013812 10.1016/j.ydbio.2003.12.004

[39] Liu Q. , Lv H., Jiang R. hicGAN infers super resolution Hi-C data with generative adversarial networks. Bioinformatics. 2019; 35:i99–i107. 31510693 10.1093/bioinformatics/btz317 PMC6612845

[40] Liu Q. , Zeng W., Zhang W., Wang S., Chen H., Jiang R., Zhou M., Zhang S. Deep generative modeling and clustering of single cell Hi-C data. 2022; bioRxiv doi:20 July 2022, preprint: not peer reviewed 10.1101/2022.07.19.500573. 36458445

[41] Frenkel B. , Montecino M., Stein J.L., Lian J.B., Stein G.S. A composite intragenic silencer domain exhibits negative and positive transcriptional control of the bone-specific osteocalcin gene: promoter and cell type requirements. Proc. Natl. Acad. Sci. U.S.A. 1994; 91:10923–10927. 7971985 10.1073/pnas.91.23.10923 PMC45138

[42] Wu M. , Zeng W., Liu W., Lv H., Chen T., Jiang R. Leveraging multiple gene networks to prioritize GWAS candidate genes via network representation learning. Methods. 2018; 145:41–50. 29874547 10.1016/j.ymeth.2018.06.002

[43] Gallagher M.D. , Chen-Plotkin A.S. The Post-GWAS era: from association to function. Am. J. Hum. Genet. 2018; 102:717–730. 29727686 10.1016/j.ajhg.2018.04.002 PMC5986732

[44] Kaukonen M. , Quintero I.B., Mukarram A.K., Hytonen M.K., Holopainen S., Wickstrom K., Kyostila K., Arumilli M., Jalomaki S., Daub C.O. et al. A putative silencer variant in a spontaneous canine model of retinitis pigmentosa. PLoS Genet. 2020; 16:e1008659. 32150541 10.1371/journal.pgen.1008659 PMC7082071

[45] Oldoni F. , Palmen J., Giambartolomei C., Howard P., Drenos F., Plagnol V., Humphries S.E., Talmud P.J., Smith A.J. Post-GWAS methodologies for localisation of functional non-coding variants: ANGPTL3. Atherosclerosis. 2016; 246:193–201. 26800306 10.1016/j.atherosclerosis.2015.12.009 PMC4773290

